# Identification of microsatellites and their effect on economic traits of Texel × Kazakh sheep

**DOI:** 10.3389/fvets.2025.1583625

**Published:** 2025-05-29

**Authors:** Yang Cunming, Bin Li, Zhu Mengting, Yiming Sulaiman, Sangang He, Mingjun Liu

**Affiliations:** ^1^College of Animal Science, Xinjiang Agricultural University, Urumqi, China; ^2^College of Animal Science and Technology, Shihezi University, Shihezi, China; ^3^Institute of Biotechnology, Xinjiang Academy of Animal Science, Key Laboratory of Genetic Breeding and Reproduction of Herbivorous Livestock, Ministry of Agriculture and Rural Affairs, Xinjiang Key Laboratory of Animal Biotechnology, Urumqi, China

**Keywords:** relationship, microsatellite locus markers, genetic diversity, Texel × Kazakh sheep, economic traits

## Abstract

**Introduction:**

Mutton has the advantages of delicious taste, high nutrition, and easy digestion. It is important to improve the production and quality of mutton in mutton sheep breeding. Microsatellite locus marker-assisted breeding is widely used to breed excellent traits of various species. It is important to search for microsatellite markers related to the economic traits (mutton production and fat content) of mutton sheep.

**Methods:**

This study aimed to explore the relationship between 11 microsatellite loci of Texel × Kazakh sheep and 12 economic traits and to seek potential loci related to the mutton production (PW: Pre-slaughter weight, CW: Carcass weight, TAW: Total breast and abdomen weight, TLT: Total weight of left anterior tendon, TLL: Total weight of left hip and leg, LD: Longissimus dorsi, OMA: Ocular muscle area) and fat deposition levels (TFW: Tail fat weight, MFW: Mesenteric fat weight, KFW: Kidney fat weight, BFT: Back fat thickness and GR: GR value) of mutton sheep.

**Results:**

Genetic analysis of the 108 Texel × Kazakh sheep hybrid population revealed 81 alleles across all loci, with a mean number of alleles (MNA) of 7.364. The population exhibited moderate observed heterozygosity (*Ho* = 0.610), high expected heterozygosity (*He* = 0.785), and substantial polymorphism (polymorphism information content, *PIC* = 0.759), indicating robust genetic diversity. Notably, the AMEL locus demonstrated significant associations with MFW (η^2^ = 0.319) and KFW (η^2^ = 0.347), while the INRA023 locus influenced CW (η^2^ = 0.260) (*adjusted p < 0.05*). No other loci showed statistically significant trait correlations after multiple-testing correction. The HH genotype at AMEL and AD genotype at INRA023 emerged as pivotal molecular markers, collectively explaining 26.0–34.7% of phenotypic variance in meat yield traits.

**Discussion:**

These findings establish a theoretical framework for precision breeding strategies, offering actionable solutions to enhance meat productivity in ovine populations through marker-assisted selection (MAS).

## 1 Introduction

As the population expands and living conditions improve, mutton has emerged as a crucial component of the human diet. Compared to other meats, mutton offers not just a delightful flavor but also greater nutritional benefits and is more readily digestible and assimilable (1, 2). Xinjiang boasts the highest per capita mutton consumption rate nationwide (3). Given the economic importance of mutton production, molecular breeding techniques like microsatellite marker-assisted selection have become crucial for genetic improvement. In eukaryotic genomes, microsatellite markers consist of brief, tandem repeat sequences that are uniformly dispersed. They exhibit significant polymorphism across individuals and demonstrate abundant distribution. Their application is extend to various domains such as genetic diversity analysis genetic diversity in populations and pinpointing molecular sites for enhancing traits (4–7). Presently, research on genetic diversity has been conducted on various species, including poultry, sheep, goats, cattle, buffaloes, and reindeer (8–11). The production of mutton and fat deposition levels in sheep directly impact their economic value. The technique of microsatellite-marker-assisted selection breeding has been utilized to enhance meat yield and fat levels in diverse animal breeds. As an illustration, the genetic variation in the ADL0019 microsatellite locus markedly influences the body mass of Hyogo-Ajidori chickens when they are 16 weeks old (12). Researchers identified significant associations between mutton production and seven microsatellite markers in Santa INÊS and hybrid sheep (13). There was a notable correlation between a polymorphism in the microsatellite locus of the Six1 gene’s promoter region in the Pietrain × Duroc × Landrace × Yorkshire pig population and factors like weaning weight, carcass weight, and thoracolumbar back fat (14). There was a notable correlation between the BM1500 microsatellite locus and various fat levels (rib fat, average fat, great fat, and marbling) in beef cattle (15). Consequently, pinpointing microsatellite loci linked to the economic traits of mutton sheep holds significant importance for mutton sheep breeding programs.

The Texel × Kazakh sheep merges the benefits of both breeds, offering advantages like robust resistance to diseases, rapid growth, superior mutton quality, and a high rate of mutton production. This hybrid is extensively reared in Xinjiang (16, 17). Consequently, using the Texel × Kazakh sheep as a model is apt for analyzing the economic traits of mutton sheep in Xinjiang. The study aims to investigate genetic variations the genetic variation of 11 microsatellite sites within the Texel × Kazakh sheep group and to explore the relationship between each genetic variant of these sites and economic traits like mutton production (PW, CW, TAW, TLT, TLL, LD, OMA) and fat deposition levels (TFW, MFW, KFW, BFW, GR). The goal is to pinpoint the microsatellite sites linked to mutton production characteristics and fat levels in Texel × Kazakh sheep, offering a theoretical foundation and technical aid for enhancing mutton production.

## 2 Materials and methods

### 2.1 Animals and sample preparation

All experimental procedures and animal ethics were approved by the Institute of Biotechnology, Xinjiang Academy of Animal Sciences (Xinjiang, China, Approval No. JXM-KX-20180304). The experimental animals in this study were sourced from the Sheep Breeding Laboratory at the Xinjiang Academy of Animal Sciences, with a total of 108 sheep (50 rams and 58 ewes). All sheep were healthy, disease-free, and raised under consistent conditions. Researchers performed sampling and measurements at the Sheep Breeding Laboratory at the Xinjiang Academy of Animal Sciences. After collection from sheep, ear tissue samples were immediately placed into, and stored at −20°C for subsequent DNA extraction. All sheep were slaughtered, and 12 traits related to mutton production and fat deposition levels were measured, including mutton production (PW, CW, TAW, TLT, TLL, LD, OMA) and fat deposition levels (TFW, MFW, KFW, BFW, GR). The specific statistical results are presented in Table 1.

**Table 1:** Production data of the 108 Texel × Kazakh sheep.

Trait	Parameter code	Name	Sample	Minimum	Maximum	Mean ± standard error
Mutton production	PW	Pre-slaughter weight/kg	108	25.10	55.25	37.82 ± 0.65
	CW	Carcass weight/kg	108	11.55	30.55	19.73 ± 0.38
	TAW	Total breast and abdomen weight/kg	108	0.54	3.01	1.54 ± 0.05
	TLT	Total weight of Left anterior tendon /kg	108	0.19	0.90	0.34 ± 0.01
	TLL	Total weight of Left hip leg/kg	108	1.09	3.92	2.60 ± 0.05
	LD	Longissimus dorsi/mm	108	193.00	543.50	360.73 ± 7.35
	OMA	Ocular muscle area /cm^2^	108	7.16	18.15	12.88 ± 0.23
Fat deposition level	TFW	Tail fat weight /g	108	0.00	1487.00	429.03 ± 25.64
	MFW	Mesenteric fat weight/g	108	50.00	1494.00	476.80 ± 30.67
	KFW	Kidney fat weight/g	108	64.10	965.50	352.90 ± 18.69
	BFT	Back fat thickness weight/mm	108	1.05	8.79	3.99 ± 0.18
	GR	GR value/mm	108	7.97	32.60	18.98 ± 0.51

### 2.2 Genomic DNA extraction

DNA was extracted from sheep ear tissues using the QIAamp 96 DNA QIAcube HT Kit (Cat.#51,331; QIAGEN, Hilden, Germany). Genomic DNA integrity was then examined by 1.0% agarose gel electrophoresis. DNA concentration was determined by a NanoDrop ND-2000 Spectrophotometer (NanoDrop Technologies, Wilmington, DE, United States). Qualified samples demonstrating OD260/OD280 ratios of 1.8–2.0 were selected were diluted to a concentration of 50 ng/μL and stored at −20°C for future use in further genotype analysis.

### 2.3 Selection and genotyping of microsatellite loci

In this study, 11 sheep microsatellite loci with high genetic diversity and amplification were selected based on recommendations from the amplification results of the International Society for Animal Genetics (ISAG)^1^ and the Food and Agriculture Organization of the United Nations (FAO).^2^ Using the DNA of 10 Texel × Kazakh sheep as a template, 11 pairs of microsatellite primers were subjected to gradient PCR tests, setting the annealing temperature gradient between 50°C and 60°C to determine optimal annealing temperatures and PCR amplification conditions for the microsatellite loci. Primers were custom-synthesized by Sangon Biotech (Shanghai, China); the primer information is shown in Table 2.

**Table 2:** Microsatellite labeled primer sequences.

Loci	Primer sequence (5′ → 3′)	Annealing temperature/°C	Product length/bp
AMEL	F:CAGCCAAACCTCCCTCTGC	58	X and Y
	R:CCCGCTTGGTCTTGTCTGTTGC		
CSRD247	F:GGACTTGCCAGAACTCTGCAAT	58	209–255
	R:CACTGTGGTTTGTATTAGTCAGG		
ETH152	F:TACTCGTAGGGCAGGCTGCCTG	56	186–200
	R:GAGACCTCAGGGTTGGTGATCAG		
INRA005	F:TTCAGGCATACCCTACACCACATG	56	125–147
	R:AAATATTAGCCAACTGAAAACTGGG		
INRA006	F:AGGAATATCTGTATCAACCGCAGTC	58	110–132
	R:CTGAGCTGGGGTGGGAGCTATAAATA		
INRA023	F:GAGTAGAGCTACAAGATAAACTTC	52	194–216
	R:TAACTACAGGGTGTTAGATGAACTC		
INRA063	F:GACCACAAAGGGATTTGCACAAGC	58	169–201
	R:AAACCACAGAAATGCTTGGAAG		
INRA172	F:CCAGGGCAGTAAAATGCATAACTG	58	126–160
	R:GGCCTTGCTAGCCTCTGCAAAC		
MAF214	F:AATGCAGGAGATCTGAGGCAGGGACG	58	189–265
	R:GGGTGATCTTAGGGAGGTTTTGGAGG		
MCM527	F:GTCCATTGCCTCAAATCAATTC	58	164–170
	R:AAACCACTTGACTACTCCCCAA		
OARFCB20	F:GGAAAACCCCCATATATACCTATAC	56	87–113
	R:AAATGTGTTTAAGATTCCATACATGTG		

Amplified using primers on the SensoQuest LabCycler, as recommended by the ISAG. The total volume of the PCR reaction system was 25 μL, which consisted of 12.5 μL of 2 × Easy Taq Super Mix, 1 μL of template DNA, 0.5 μL each of the upstream and downstream primers at a concentration of 10 μmol·L^−1^, and 10.5 μL of ddH₂O. The PCR amplification procedure was as follows: pre-denaturation at 95°C for 10 min; denaturation at 95°C for 40 s, annealing at the optimal annealing temperature for 30 s, and extension at 72°C for 60 s, with a total of 30 cycles; extension at 72°C for 10 min; and storage at 4°C. After the PCR products were detected by 2.0% agarose gel electrophoresis, genotyping was performed using the Fragment Analyzer™ automated capillary electrophoresis instrument, and the allele results were read using PROSize 3.0.

### 2.4 Data statistical analysis

After the original data was organized using Microsoft Excel 2016, individuals showing ≥4 validated genotypes, and R 4.3.1 software was used to analyze data such as the number of alleles (Na), observed heterozygosity (Ho), expected heterozygosity (He), and the polymorphism information content (PIC) of the microsatellite loci.

Genetic association analyses were performed on the R statistical platform (version 4.3.1) following this workflow: (1) Linear model construction: Generalized linear models (GLM) were implemented using the lm() function to assess genotype effects on phenotypic traits, with the model formula specified as:


$$Y_{ijk}=\mu +G_{i}+S_{j}+e_{jlk}$$

In the formula: *Y_ijk_* represents the individual phenotypic value, *μ* represents the population mean value, *G_i_* represents the fixed effect of the individual genotype, *S_j_* represents the individual sex effect, and *e_ijk_* represents the random residual effect.

(2) Hypothesis testing and correction: Analysis of variance (ANOVA) was conducted using the anova() function to evaluate genotype main effects. Raw *p*-values were adjusted by Bonferroni correction (correction factor = 132, corresponding to the number of locus × trait combinations) to control family-wise error rate in multiple testing; (3) Effect size estimation: The η^2^ values quantifying the proportion of phenotypic variance explained by genotypes (range 0–1) were computed using the eta_squared() function from the effect size package, Small effect: η^2^ < 0.01;Medium effect: 0.01 ≤ η^2^ < 0.14;Large effect: η^2^ ≥ 0.14 (18); (4) Post-hoc multiple comparisons: For loci passing the significance threshold (adjusted *p* < 0.05), pairwise comparisons were performed using the least significant difference (LSD) method implemented in the LSD.test() function from the agricolae package. Results were presented as “mean ± standard deviation” to demonstrate inter-group differences; (5) Statistical significance thresholds were defined as: *p < 0.05, **p < 0.01.

## 3 Results

### 3.1 Genomic DNA detection and electrophoresis typing

After the DNA extracted from the tissue samples was detected by 1.0% agarose gel electrophoresis, the DNA bands were clear. After the PCR amplification products were detected by 2.0% agarose gel electrophoresis, there were no obvious miscellaneous bands, and no primer dimers were generated, which could be used for subsequent experiments. Allele typing was performed using the Fragment Analyzer™ automated capillary electrophoresis system, and alleles were read using PROSize 3.0. The results demonstrate that the peak patterns in the electrophoresis diagrams were fairly distinct and complete. The typing results are shown in Figure S1.

### 3.2 Analysis of microsatellite genetic diversity

As presented in Tables 3, a total of 81 alleles were detected across 11 microsatellite loci in the Texel × Kazakh sheep population. The AMEL, CSRD247 and MAF214 locus exhibited the highest number of alleles (Na = 9), while ETH152 and MCM527 showed the lowest (Na = 5), with a mean of 7.364 alleles per locus. The observed heterozygosity (Ho) ranged from 0.385 to 0.862, with the MAF214 locus displaying the lowest value (Ho = 0.385) and the INRA172 locus the highest (Ho = 0.862), yielding an average Ho of 0.610. The expected heterozygosity (He) varied between 0.608 and 0.51, where the CSRD247 locus recorded the minimum (He = 0.608) and the MAF214 locus the maximum (He = 0.851), resulting in a mean He of 0.785. The polymorphic information content (PIC) values spanned from 0.589 to 0.834, with the CSRD247 locus showing the lowest PIC (0.589) and the MAF214 locus the highest (0.834), averaging 0.759 across all loci. All microsatellite loci demonstrated high polymorphism (PIC > 0.5). These results confirm that the 11 microsatellite markers are polymorphic and suitable for evaluating genetic diversity in Texel × Kazakh sheep.

**Table 3:** Genetic polymorphism information of the 11 microsatellite loci.

Loci	N	Na	Ho	He	PIC
AMEL	108	9	0.551	0.811	0.787
CSRD247	108	9	0.685	0.608	0.589
ETH152	108	5	0.667	0.757	0.718
INRA005	108	8	0.693	0.833	0.811
INRA006	108	7	0.66	0.79	0.762
INRA023	108	8	0.563	0.816	0.793
INRA063	108	8	0.565	0.841	0.821
INRA172	108	6	0.862	0.794	0.765
MAF214	108	9	0.385	0.851	0.834
MCM527	108	5	0.667	0.764	0.725
OARFCB20	108	7	0.411	0.775	0.746
Mean	108	7.364	0.61	0.785	0.759

### 3.3 Association analysis between microsatellite loci and economic traits

As presented in Table 4, microsatellite-based association analysis revealed significant genetic influences: The AMEL locus demonstrated substantial effects on both MFW (adjusted *p* = 0.043) and KFW traits (adjusted *p* = 0.010), while the INRA023 locus was significantly associated with CW (adjusted *p* = 0.044). No other loci showed statistically significant correlations with the target traits after multiple testing correction.

**Table 4:** Decoding the genetic influence of AMEL and INRA023 microsatellites on MFW, KFW, and CW Traits.

Traits	Loci	Genetype	HH	GG	II	DI	GI	JJ	AG	BH	CI	DJ	CH	CF	Adjusted-P	η^2^
AMEL	MFW	Mean	634.6	629.83	552.2	487.86	484.5	456.67	419	412.5	241.38	220.43	197	162.5	0.043	0.319
		SD	325.4	273.31	340.59	333.73	208.17	334.79	329.26	227.91	134.61	73.08	164.78	146.09		
		Sig	a	a	a	ab	ab	ab	ab	ab	b	b	b	b		
	KFW	Genetype	HH	JJ	GG	GI	DI	AG	II	BH	DJ	CI	CF	CH	Adjusted-P	η^2^
		Mean	492	458.25	392.64	358.88	345.51	334.4	311	227	225.57	223.75	161.62	148	0.01	0.347
		SD	179.97	236.05	203.8	126.22	215.38	187.78	176.45	103.08	102.96	93.95	76.44	47.06		
		Sig	a	ab	abc	abcd	bcd	bcd	bcd	cd	d	d	d	d		
INRA023	CW	Genetype	AD	CH	AF	EH	CC	BH	GG	EE	BB				Adjusted-P	η^2^
		Mean	26.36	21.24	20.12	20.07	19.67	19.15	18.98	18.62	17.49				0.044	0.26
		SD	2.99	4.22	4.16	3.75	3.29	2.17	3.99	2.47	4.86					
		Sig	a	b	bc	bc	bc	bc	bc	c	c					

Post quality control, the AMEL locus comprised 12 genotypes for MFW, with phenotypic values ranging from 162.5 ± 146.09 (CF) to 634.6 ± 325.4 (HH). High-performance genotypes (HH, GG, II; mean range: 634.6–552.2) exhibited significantly elevated phenotypic values compared to low-performance genotypes (CI, DJ, CH, CF; 241.38–162.5; *p* < 0.05). Intermediate genotypes (DI, GI, JJ, AG, BH; 487.86–412.5) showed no significant differentiation from either extreme group (*p* > 0.05), accounting for 31.9% of phenotypic variance (η^2^ = 0.319). For KFW, phenotypic values spanned 148.0 ± 47.06 (CH) to 492.0 ± 179.97 (HH). The HH genotype (492.0) significantly outperformed low-value genotypes (BH, CH, DJ; 227.0–148.0; *p* < 0.05) but displayed comparable performance to intermediate genotypes (JJ, GG, GI; 458.25–358.88), with stronger genetic regulation (η^2^ = 0.347).

The INRA023 locus, associated with CW (adjusted *p* = 0.044), contained 9 genotypes showing phenotypic variation from 17.49 ± 4.86 (BB) to 26.36 ± 2.99 (AD). The AD genotype demonstrated superior phenotypic expression, significantly surpassing all other genotypes (*p* < 0.05). The CH genotype (21.24 ± 4.22) exhibited a marginal yet significant advantage over the lowest-performing group (EE, BB; 18.62–17.49; p < 0.05), whereas intermediate genotypes (AF, EH, CC, BH, GG; 20.12–18.98) lacked intra-group differentiation, reflecting moderate genetic control (η^2^ = 0.260).

Both loci exhibited large effect sizes (η^2^ > 0.26) across three economically critical traits, exceeding the conventional threshold for substantial genetic influence (Cohen’s benchmark: η^2^ > 0.14). The HH genotype at AMEL and AD genotype at INRA023 emerged as pivotal molecular markers for meat sheep breeding, explaining 26.0–34.7% of phenotypic variance. These results establish a robust framework for functional genomics investigations and precision breeding strategies.

## 4 Discussion

Genetic improvement of livestock is inseparable from abundant genetic variation, and the genetic diversity of microsatellite loci can well reflect the genetic variation status of a population (19).

The study selected 11 microsatellite loci to analyze the genetic diversity of the Texel × Kazakh sheep population. As can be seen from the genetic diversity parameters such as the number of alleles, heterozygosity, and polymorphic information content shown in Table 3, the Texel × Kazakh sheep population has a relatively high level of genetic diversity. All the 11 microsatellite loci selected in this study had more than 4 alleles, with an average number of alleles being 7.364. The mean NA value of the Texel × Kazakh sheep population was lower than that of 14 sheep breeds in Iran (19), five Turkish sheep breeds (Gökçeada, Kıvırcık, Karacabey Merino, Sakız, and Pırlak) (MeanNA: 11.89) (20, 21), five Kazakhstani sheep populations (MeanNA: 13.416) (22), seven Montenegrin sheep breeds (MeanNA: 13.5) (23), and 11 native sheep breeds in India (24), but higher than the three populations of Kari sheep (25) and Nellore sheep (26). The magnitude of the expected heterozygosity (He) of a population can be attributed to the number of alleles detected at the selected microsatellite loci (27). In the results of this study, the He values were all greater than 0.6, and the average He value was 0.785, which was similar to the results of 24 sheep populations in Turkey (20, 21), Kazakhstan (22) and Iran (19), and higher than that of 30 sheep populations in Montenegro (23), Croatia and Bosnia and Herzegovina (28) and India (25), as well as three populations of Kari sheep (25), Nellore sheep (26) and China hu sheep (29). Among the 11 markers, the Ho values of 10 markers were greater than 0.5, and the mean Ho value was 0.610, which was relatively low compared with the results of other studies (19–26, 28). The polymorphic information content (PIC) usually reflects the polymorphism of microsatellite loci. When the PIC value is greater than 0.5, the population is considered to be highly polymorphic, and when the PIC value is greater than 0.7, the microsatellite locus is regarded as a relatively good locus (30–32). In this study, the PIC values of 10 out of the 11 loci were greater than 0.7, and the average PIC value was 0.759, indicating that the Texel × Kazakh sheep population in this study had a high level of polymorphism and that the selected 11 microsatellite loci were suitable for the correct assessment of the genetic diversity of the Texel × Kazakh sheep population and the association analysis with economic traits. The PIC values in the results of this study were higher than those reported by Ibrahim et al. (25), Jeyakumar and Ramachandran (26), and Sun et al. (29), similar to the results reported by Öner et al. (20), Dossybayev et al. (22), and Marković et al. (23), but lower than the results of Vajed Ebrahimi et al. (19) and Yilmaz et al. (21).

Microsatellite marker-assisted selection for economic traits has been applied in many kinds of animals (9, 33, 34), and the amount of mutton production directly determines the economic benefits of mutton sheep. For example, Tatsuda (12) found in their study that the body weight at 16 weeks of age of the DD genotype at the ADL0019 microsatellite locus in Hyogo-Ajidori chickens was higher than that of the CC genotype. Petroli et al. (13) showed that seven microsatellite markers in SANTA INÊS AND CROSSBRED sheep were significantly associated with the mutton production of sheep. Wu et al. (14) found that there was a microsatellite locus in the promoter region of the Six1 gene in the Pietrain × Duroc × Landrace × Yorkshire pigs population, and its polymorphism was significantly associated with weaning weight, carcass weight, and thoracic, lumbar and dorsal fat.

Fat deposition level is one of the important production traits, and the functions of fat in different parts are different. In this study, a total of five traits were focused on, including tail fat weight, mesenteric fat weight, kidney fat weight, back fat thickness, and GR value. Fitzsimmons et al. (15) found in their study that the 139 bp allele at the BM 1500 microsatellite locus could significantly increase rib fat, average fat, great fat, and marbling, and reduce the lean meat percentage of ribs, while the effect of the 147 bp allele was the opposite. Wu et al. (35) found that the SJ158 gene locus in the CA3 gene of Yorkshire × Meishan pigs was significantly associated with fat percentage, lean meat percentage, visceral fat percentage, backfat thickness at the 6th–7th thoracic vertebrae and backfat thickness at the buttocks. Compared with pigs of other genotypes, pigs with the AA genotype had the highest lean meat percentage, the lowest fat percentage, and slaughter percentage, while pigs with the BC genotype had the highest fat percentage, visceral fat percentage, and backfat thickness. Allele A of Yorkshire pigs was related to an increase in lean meat percentage and rib number and a decrease in fat percentage.

The INRA023 locus, recommended by the International Society for Animal Genetics (ISAG) as a microsatellite marker for sheep, exhibits high polymorphism and is commonly employed in genetic diversity analysis and parentage verification. Dossybayev et al. (22) identified 16 alleles at this locus in Kazakh sheep populations, demonstrating exceptional polymorphism (Polymorphism Information Content, PIC = 0.8654). Similar findings were reported by Isakova et al. (36) in Kyrgyzstan Mountain Merino sheep. In contrast, the AMEL locus, localized to sex chromosomes, has been utilized as an auxiliary marker for genetic diversity studies due to its sequence conservation. Oner et al. (37) observed that the AMEL locus in Turkish native sheep breeds exhibits Y-chromosomal sequence conservation without polymorphism, primarily serving as a sex-specific marker. Notably, prior studies have not established associations between the INRA023 and AMEL loci with economically significant traits.

Our results revealed substantial genetic effects (large effect sizes) for the AMEL locus on MFW and KFW, and for the INRA023 locus on carcass width (CW). Specifically, the HH genotype at the AMEL locus and the AD genotype at the INRA023 locus emerged as pivotal candidate markers for meat sheep breeding.

## 5 Conclusion

This study identified 11 microsatellite loci exhibiting high genetic diversity in the Texel × Kazakh sheep hybrid population. Notably, the AMEL locus (associated with Meat Fat Weight, MFW and Kidney Fat Weight, KFW) and INRA023 locus (associated with Carcass Width, CW) demonstrated significant correlations with meat production traits and adiposity. Substantial genetic effects were observed for the AMEL locus on MFW (η^2^ = 0.319) and KFW (η^2^ = 0.347), as well as for the INRA023 locus on CW (η^2^ = 0.260). The HH genotype at the AMEL locus and the AD genotype at the INRA023 locus emerged as pivotal candidate markers for meat sheep breeding programs. These findings provide a theoretical foundation for optimizing breeding strategies and trait improvement in meat sheep, while establishing a technical framework to address challenges in enhancing meat yield productivity.

## Data Availability

The datasets presented in this study can be found in online repositories. The names of the repository/repositories and accession number(s) can be found in the article/Supplementary material.

## Footnotes

^1^ https://www.isag.us

^2^ http://www.fao.org
