# Supplementary material for: Identification of microsatellites and their effect on economic traits of Texel × Kazakh sheep
Source: Front Vet Sci. 2025 May 29;12:1583625. doi: 10.3389/fvets.2025.1583625 (PMC12180305; doi:10.3389/fvets.2025.1583625)
Supplement: Supplementary file 12 [file Image_1.pdf]

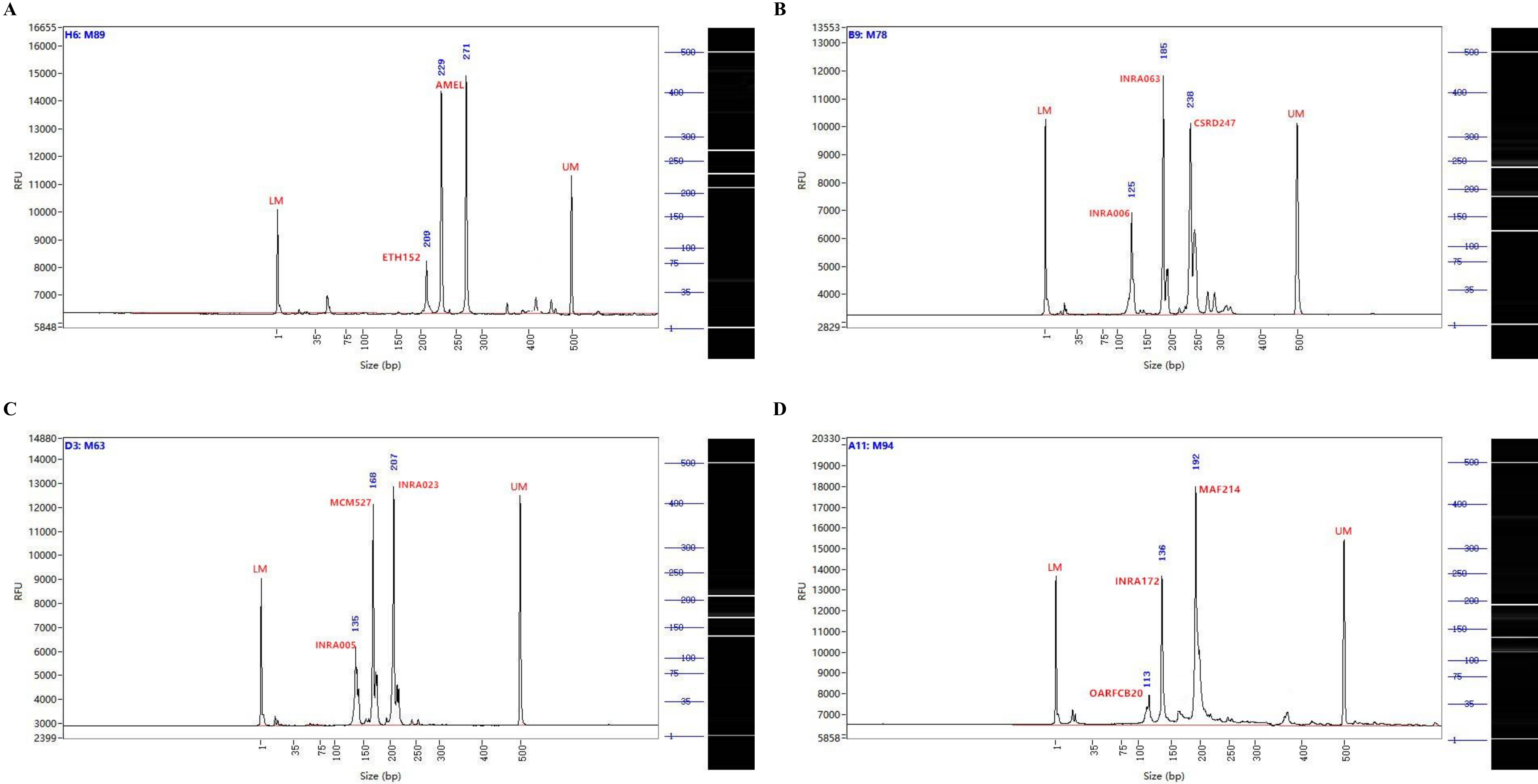

Supplementary Figure S1. Results of 11 microsatellite loci in different individuals: Panels A - D sequentially display a total of 11 microsatellite loci, namely ETH152, AMEL, INRA006, INRA063, CSRD247, INRA005, MCM527, INRA023, OARFCB20, INRA172 and MAF214.
